# Nanocurcumin Attenuates Doxorubicin‐Induced Cardiotoxicity by Activating the Wnt/β‐Catenin/GSK‐3β Signaling Axis in Wistar Rats

**DOI:** 10.1002/jbt.71158

**Published:** 2026-11-04

**Authors:** Noha A. Alshuwayer, Qamraa H. Alqahtani, Marwa H. Hussein, Raeesa Mohammed, Abdulaziz Siyal, Iman H. Hasan

**Affiliations:** ^1^ Department of Anatomy, College of Medicine King Saud University Riyadh Saudi Arabia; ^2^ Department of Pharmacology and Toxicology, College of Pharmacy King Saud University Riyadh Saudi Arabia

**Keywords:** cardiotoxicity, doxorubicin, nanocurcumin, Wnt/β‑catenin–GSK‑3β

## Abstract

Doxorubicin (DOX)–induced cardiotoxicity remains a major therapeutic limitation, emerging at the intersection of oxidative stress, mitochondrial impairment, and disruption of survival signaling pathways. The Wnt/β‑catenin–GSK‑3β pathway is an essential regulator of cardiomyocyte survival. To assess the cardioprotective potential of nanocurcumin (N‐Cur) in this condition, twenty‐four Wistar rats were divided into four groups (N‐Cur; 80 mg/kg, oral) was given for 2 weeks, while DOX was administered on day seven as a single dose. N‐Cur treatment significantly standardized DOX‑induced elevations in cTnI and CK‑MB, restored cardiac antioxidants (GSH, SOD), decreased MDA levels with *p* ≤ 0.01, significantly restored Nrf2, and reduced pro‑inflammatory mediators (TNF‑α, IL‑6, NF‑κB‑p65) (*p* < 0.001), thereby augmenting its twin roles as a potent anti‐inflammatory and antioxidant agent. The histopathological changes and inflammatory infiltration caused by DOX were significantly diminished following N‐Cur treatment. Further, DOX inhibited Wnt/β‑catenin signaling and increased GSK‑3β expression, whereas N‐Cur reversed these changes in immunohistochemistry studies. Collectively, these findings indicate that N‐Cur provides significant cardiomyocyte protection and efficiently modifies the Wnt/β‑catenin–GSK‑3β axis.

## Graphical abstract

Nanocurcumin (N‐Cur) mitigates Doxorubicin (DOX)‐induced cardiotoxicity by suppressing oxidative stress, inflammation, and histopathological damage in Wistar rats. Mechanistically, N‐Cur restores cardiomyocyte survival by activating the Wnt/β‐catenin signaling pathway and downregulating GSK‐3β expression.

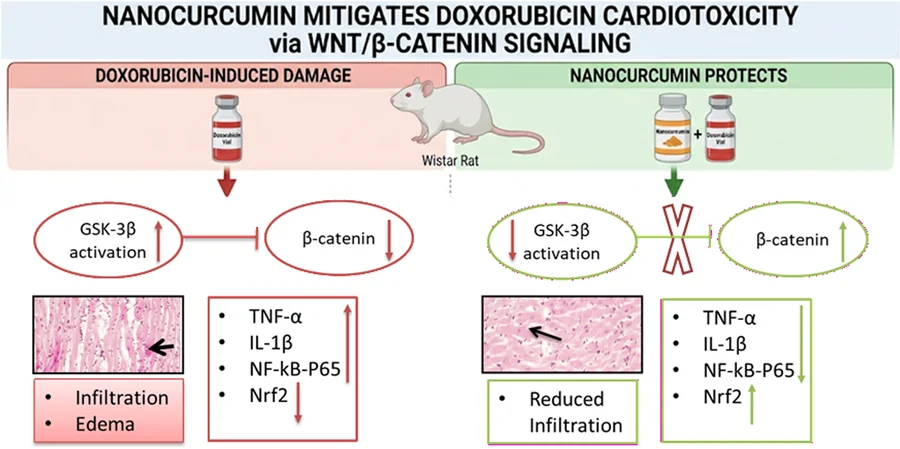

## 1 Introduction

Doxorubicin (DOX) is an anthracycline chemotherapeutic agent originally isolated from the bacterium *Streptomyces peucetius*. It is extensively employed in the management of numerous malignancies; however, its therapeutic utility is substantially constrained by dose‐limiting cardiotoxicity, a predominant long‐term adverse effect that contributes significantly to morbidity and mortality among cancer survivors [1]. The pathogenic cascade is driven predominantly by oxidative stress and inflammatory signaling, with multiple regulated cell‑death pathways contributing to DOX‑induced cytotoxicity [2] Multiple studies suggest that dysregulation of the Wnts/β‑catenin pathway and its upstream modulator, glycogen synthase kinase‑3β (GSK‑3β), considerably contributes to the underlying molecular process responsible for DOX‐induced cardiotoxicity [3, 4].

The Wnt/β‑catenin signaling pathway is a fundamental regulator of cellular survival and cardiac remodeling. Under physiological conditions, Wnt signaling is almost inactive, and β‑catenin is continuously phosphorylated by GSK‑3β, targeting it for ubiquitination and subsequent proteasomal degradation. Under pathological conditions, Wnt signaling is activated to participate in the tissue repair process. The inhibitory effect on GSK‑3β prevents β‑catenin phosphorylation, thereby facilitating its stabilization, cytoplasmic accumulation, and subsequent nuclear translocation [5]. It also contributes to DOX ‑induced cardiotoxicity by directing mitochondrial dysfunction and apoptotic signaling, besides its crosstalk with other cellular stress pathways [6].

In addition, the Nrf‐2 (nuclear factor‐erythroid factor 2‐related factor 2) signaling pathway is activated. This pathway was linked to Wnt signaling in hepatocytes, where Wnt‐3a disrupted GSK‐3β phosphorylation and ubiquitination of Nrf‐2 [7, 8].

DOX has been shown to suppress Wnt/β‐catenin signaling, contributing to cardiomyocyte injury; it reduces β‑catenin levels and diminishes pathway activation, impairing mitochondrial integrity and supporting apoptotic signaling [9]. In addition, DOX upregulates Dickkopf‑1 (DKK1), a potent inhibitor of the Wnt pathway, suppressing β‑catenin signaling and worsening myocardial injury. Remarkably, restoring β‑catenin activity mitigates DOX‑induced cardiotoxicity, confirming its protective function [10]. DOX‑induced oxidative stress further reduces Wnt/β‑catenin signaling, leading to a vicious cycle of ROS accumulation and cardiomyocyte death [11].

Numerous antioxidants were examined for their impact on DOX‐induced cardiotoxicity. In this study, we investigated the potential N‐Cur protective effect by modulating the Wnt/β‐catenin and NRF‐2 signaling pathways. Curcumin is believed to exert cardioprotection through multiple balancing mechanisms, including vigorous antioxidant activity and reduced ROS generation, thereby preserving redox balance [12]. In addition, Curcumin modulates pro‑inflammatory pathways, resulting in reduced cytokine production and attenuated inflammatory signaling [13]. Furthermore, Curcumin causes inhibition of apoptotic signaling, thus enhancing cardiomyocyte survival [14]. Nonetheless, its therapeutic potential is restricted by its low systemic bioavailability, which is noticeably overcome by N‐Cur formulations, which enrich cellular uptake and significantly improve pharmacological efficiency [15].

Curcumin has been shown to suppress GSK‑3β activity, preventing β‑catenin proteasomal degradation and stabilizing it, which promotes survival signaling, enhancing cellular resistance during oxidative and inflammatory stress [13].

Previous studies have shown that N‐Cur provides significantly greater cardioprotection than native curcumin, including improved cardiac histopathology, restoration of cardiac biomarkers, and reduction of oxidative and apoptotic damage [16]. N‐Cur enhanced performance comes from optimized physicochemical properties, greater intracellular uptake, and improved intracellular drug dynamics, all of which synergistically elevate its biological potency. This enhanced profile enables N‐Cur to more effectively modulate key regulatory pathways, including oxidative stress (Nrf2), apoptosis (caspase and Bcl‑2 family signaling), inflammation (NF‑κB), and major pro‑survival cascades such as PI3K/Akt and potentially Wnt/β‑catenin [17, 18].

While these previous studies are consistent, direct experimental evidence that N‐Cur modulates the Wnt/β‑catenin pathway specifically in DOX‑induced cardiotoxicity remains scarce. This scientific gap highlights the need for in‐depth investigations to analyze this signaling pathway in cardiac tissue following N‐cur treatment.

In this study, N‐Cur enhanced curcumin's effects by increasing intracellular concentration and prolonging its release kinetics. Consequently, N‐Cur can efficiently restore GSK‐3β and β‐catenin to normal levels, shifting signaling toward cardioprotection.

## 2 Materials and Methods

### 2.1 Animals and Treatments

Ten‐week‐old male Wistar rats (180–200 g) were housed under standard laboratory conditions with free access to food and water, maintained at 23 ± 2°C under a 12‐h light/dark cycle for 1 week before the study. The experimental procedures were approved by the Ethics Committee of King Saud University (KSU‐SE‐24‐68).

DOX was prepared in physiological saline, while N‐Cur (Lipolife, Essex, UK) was suspended in 1% carboxymethylcellulose (CMC). Liposomal formulations incorporating patented C3 Curcumin are engineered to enhance oral bioavailability and maintain physicochemical stability through stringent quality control measures. The active complex, standardized to 95% curcuminoids, is encapsulated within phospholipid‑based liposomes derived from high‑grade lecithin, thereby shielding the curcuminoids from gastrointestinal degradation and facilitating improved systemic absorption. Manufacturing protocols consistently achieve vesicular dimensions within the sub‑micron range (approximately 100–200 nm) and maintain a polydispersity index (PDI < 0.3), parameters that collectively ensure a uniform colloidal dispersion and prolonged suspension stability. While encapsulation efficiency and zeta potential are subject to proprietary batch‑specific validation, their restricted disclosure reflects internal quality assurance practices designed to sustain both the formulation's bioavailability and long‑term stability. Animals were randomly assigned to four groups (*n* = 6 per group) as follows:

- **Group I (Control)**: received the normal saline orally for 14 consecutive days.
- **Group II (N‐Cur)**: received N‐CUR (80 mg/kg, orally) for 14 days [19].
- **Group III (DOX)**: received the normal saline orally for 14 days and a single intraperitoneal injection of DOX (15 mg/kg) on Day 7 [20].
- **Group IV (DOX** + **N‐Cur)**: received N‐CUR (80 mg/kg, orally) for 14 days along with a single intraperitoneal injection of DOX (15 mg/kg) on Day 7.

At the end of the experimental period, blood samples were collected under anesthesia for serum separation, and the animals were euthanized. Cardiac tissues were excised; portions were fixed in 10% neutral buffered formalin for histological analysis, while the remaining tissues were homogenized in Tris–HCl buffer, centrifuged, and the supernatants were stored at −80°C for biochemical assessments.

### 2.2 Biochemical Assays

Serum levels of cardiac troponin I (cTnI) and Creatine kinase‐MB (CK‐MB) activity were measured using an ELISA kit obtained from Elabscience (E‐EL‐R1253, China) and Randox (CK4043, UK), respectively.

ELISA kits were used to quantify inflammatory markers, including tumor necrosis factor‐alpha (TNF‐α), interleukin‐1β (IL‐1β), and NF‐Kb‐p65, supplied by MyBioSource (MBS733016, MBS269892, MBS287521; USA, respectively). In addition, Nrf2 levels were measured using a BT LAB ELISA kit (Cat. NO. E1083Ra, China), according to the manufacturer's protocols. Oxidative stress parameters, including malondialdehyde (MDA) levels, reduced glutathione (GSH), and superoxide dismutase (SOD) activities, were evaluated using kits from Biodiagnostic (MD2528, TA2511, and SD2521, Egypt; respectively).

### 2.3 Histopathological and Immunohistochemistry (IHC) Examination

Directly after the rats were sacrificed; three heart tissues from the control and all the treated groups were fixed in 10% formaldehyde for 24‐h. The fixed tissues were processed to prepare 5‐µm‐thick paraffin sections on ordinary glass slides, for staining with hematoxylin and eosin (H&E), to examine the structure of myocardial cells and interstitial tissue (endomysium). Other heart sections were fixed on charged glass slides to be processed for the immune‐histochemical detection of β‐Catenin and GSK‐3β. Briefly, the tissue sections mounted on slides were blocked by immersion in 3% hydrogen peroxide (H2O2) solution for 5 min. After washing in phosphate‐buffered saline (PBS; pH 7.4) for 15 min, three times, the slides were incubated with protein block (R&D; Cell and Tissue Staining Mouse Kit; CTS002) for 15 min to block the non‐specific binding of antibodies. Primary antibodies—mouse monoclonal anti β‐catenin (Cat. No. 14‐2567‐82) and GSK3 alpha/beta Monoclonal antibody (Cat. No. 44‐610) were purchased from Thermo Fisher Scientific (Waltham, USA). Tissue sections were incubated overnight at 4°C with both primary antibodies diluted at a 1:100 ratio. On the second day, the slides were incubated with a protein blocking solution for 15 min. After washing with phosphate‐buffered saline (PBS), three times. Secondary (Cat. No. RE7280‐CE) detection was carried out using the Novolink Polymer Detection System (Leica Biosystems, Newcastle, UK) according to the manufacturer's instructions. Color development was achieved using a 3,3 ‐Diaminobenzidine (DAB) detection kit. For each sample, five randomly selected high‐power fields (HPFs) were analyzed under an optical microscope at 400X magnification. Image analysis of immuno‐stained sections was performed using Image J software (version 1.32j, National Institutes of Health) (NIH, Bethesda, MD, USA), and data were expressed as a percentage relative to the control group.

### 2.4 Quantitative RT‐PCR

Changes in heart β‐catenin (CTNNB1), GSK3, and Axin2 mRNA were assayed using qRT‐PCR. RNA was isolated using Trizol (Invitrogen, Thermo Fisher Scientific, USA, Cat. no. 15596026) and RNA samples with A260/A280 value ≥ 1.8 after purification were processed for cDNA synthesis via reverse transcription. cDNA was amplified using SYBR Green (Thermo Fisher Scientific, USA; Cat. no. 4309155) and primer pairs in Table 1. Relative transcript expression levels were calculated by using the 2^−∆∆CT^ method with β‐actin as the reference control.

**Table 1:** Primer sequences for RT‐qPCR.

Genes	Primers sequences
β‐Catenin (CTNNB1) (NM_001098209)	5′CACAAGCAGAGTGCTGAAGGTG3′
	5′GATTCCTGAGAGTCCAAAGACAG 3′
GSK3 (NM_002093)	5′CCGACTAACACCACTGGAAGCT 3′
	5′AGGATGGTAGCCAGAGGTGGAT 3′
Axin2 (NM_004655)	5′GTACAGCCCGCCAACTAACA3′
	5′TCCGGATCCACCTACTCTGAA 3′
β‐Actin	5′CCTGCTTGCTGATCCACA3′
	5′CTGACCGAGCGTGGCTAG 3′

### 2.5 Statistical Analysis

Data were analyzed using one‐way analysis of variance (ANOVA) followed by Tukey's post hoc test using GraphPad Prism‐9.0 software (GraphPad Software, Boston, MA, USA). Results are presented as mean ± standard error (SE), and a value of *p* < 0.05 was considered statistically significant.

## 3 Results

### 3.1 N‐Cur Mitigates DOX‐Induced Myocardial Injury

To assess the cardioprotective potential of N‐Cur against DOX‐induced myocardial injury, serum cardiac injury markers were evaluated. Administration of DOX significantly elevated cTnI (Figure 1A) and CK‐MB (Figure 1B) levels, reflecting myocardial damage. Treatment with N‐Cur markedly reduced these elevations, resulting in significantly lower cTnI and CK‐MB levels compared with the DOX group (*p* < 0.001).

**Figure 1:**
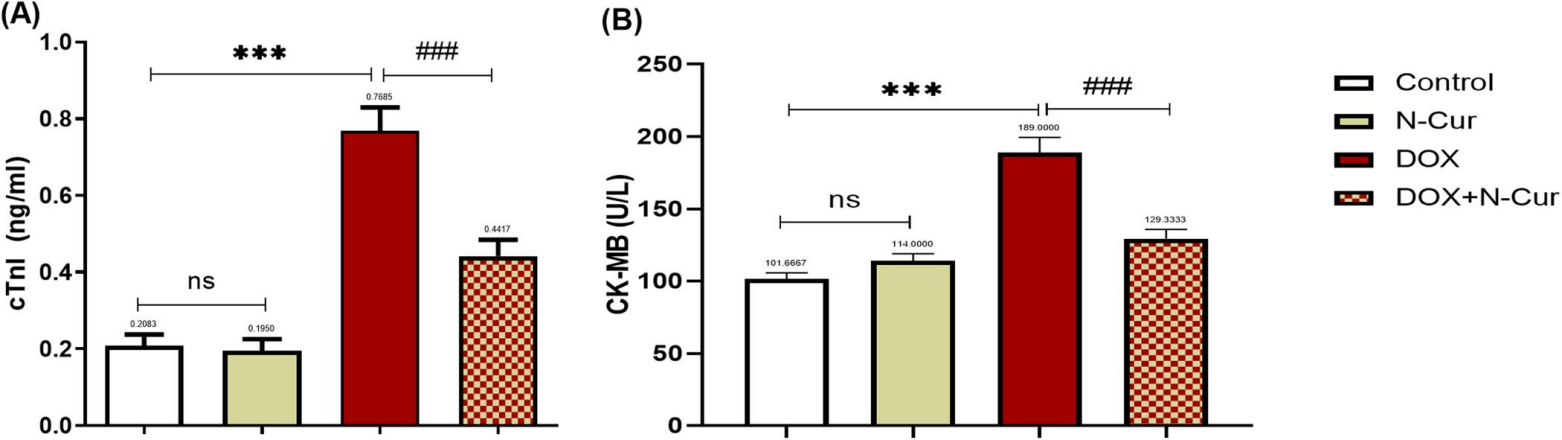
N‐Cur decreases serum levels of cTnI (A) and CK‐MB (B) in DOX‐ induced cardiac injury. Abbreviations: cTnI, cardiac troponin‐I; CK‐MB, creatine kinase; DOX, doxorubicin; N‐Cur, nanocurcumin. Data are means ± SE, (*n* = 6). *** *p* ≤ 0.001 compare to control group, and ^###^ *p* ≤ 0.001 compare to DOX group.

H&E stained sections of rat heart revealed normal myocardial architecture in both the control and N‐Cur groups (Figure 2A,B), characterized by regularly arranged cardiac fibers and preserved nuclei. In contrast, rats received DOX showed patches of sever degeneration of myocardium fibers, including cytoplasm (arrow) and nuclear apoptosis (Figure 2C). Heart section from rat administered both DOX and N‐Curcumin showed marked decrease of myocardial degenerations with few less degenerated cells (Figure 2D).

**Figure 2:**
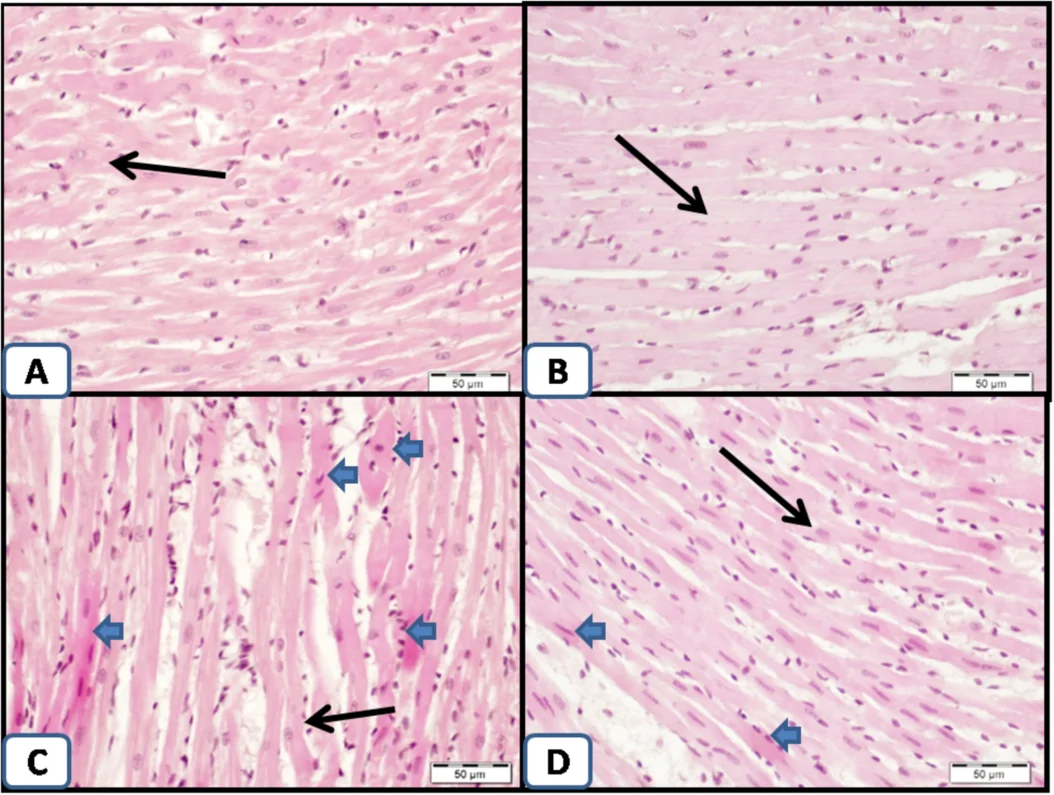
H&E‐stained sections of rat heart; scale bar 50 µm. (A) and (B) are sections from a normal control and a rat that received N‐Cur, showing normal cardiac cell fibers (arrow). (C) section from rat received DOX, producing patches of severe degeneration of myocardium cytoplasm (arrow)and nuclear apoptosis (arrow heads). (D) Heart section from a rat administered DOX and N‐Cur showing marked decrease of myocardial degenerations (arrow) with fewer degenerated cells (arrow heads).

### 3.2 N‐Cur Mitigates DOX‐Induced Oxidative Stress in the Rat Heart

Table 2 showed that DOX administration significantly elevated cardiac lipid peroxidation, as indicated by increased MDA levels, depleted endogenous antioxidants, including GSH and SOD. Treatment with N‐Cur effectively reversed these alterations, lowering MDA while restoring GSH and SOD activities (*p* < 0.001), demonstrating its potent antioxidant effect against DOX‐induced cardiac oxidative stress.

**Table 2:** N‐Cur attenuates the DOX‐induced cardiac oxidative stress.

Groups				
Parameters	Control	N‐Cur	DOX	DOX + N‐Cur
MDA (nmol/100 mg)	29.3 ± 1.35	26.7 ± 0.94	72.4 ± 2.42***	34 ± 2.55^##^
SOD (nmol/100 mg)	10.98 ± 0.24	11.03 ± 0.32	4.8 ± 0.2***	7.06 ± 0.26^##^
GSH (U/g)	25.9 ± 0.8	23 ± 0.6	12.7 ± 0.9***	17.2 ± 0.72^##^

Abbreviations: N‐Cur, nanocurcumin; DOX, doxorubicin; GSH, glutathione; MDA, malonaldehyde; SOD, superoxide dismutase. Data are means ± SE, (*n* = 6). ***p ≤ 0.001 compare to control group, and ^##^ *p* ≤ 0.01 compare to DOX group.

### 3.3 N‐Cur Mitigates DOX‐Induced Cardiac Inflammation and Oxidative Stress via Nrf2 Activation

The impact of N‐Cur on DOX‐induced myocardial inflammation was assessed via ELISA (Figure 3). DOX markedly increased TNF‐α and IL‐1β levels in cardiac tissues (*p* < 0.001; Figure 3A,B) and elevated NF‐κB p65 expression (*p* < 0.001; Figure 3D). Furthermore, DOX significantly suppressed the expression of Nrf2 (*p* < 0.001; Figure 3C), representing a breakdown in the heart's endogenous antioxidant defenses. In contrast, N‐Cur treatment significantly suppressed NF‐κB p65, restored Nrf2 expression, and reduced TNF‐α and IL‐1β levels (*p* < 0.001), confirming its dual role as a potent anti‐inflammatory and antioxidant agent in the context of DOX‐induced cardiac injury.

**Figure 3:**
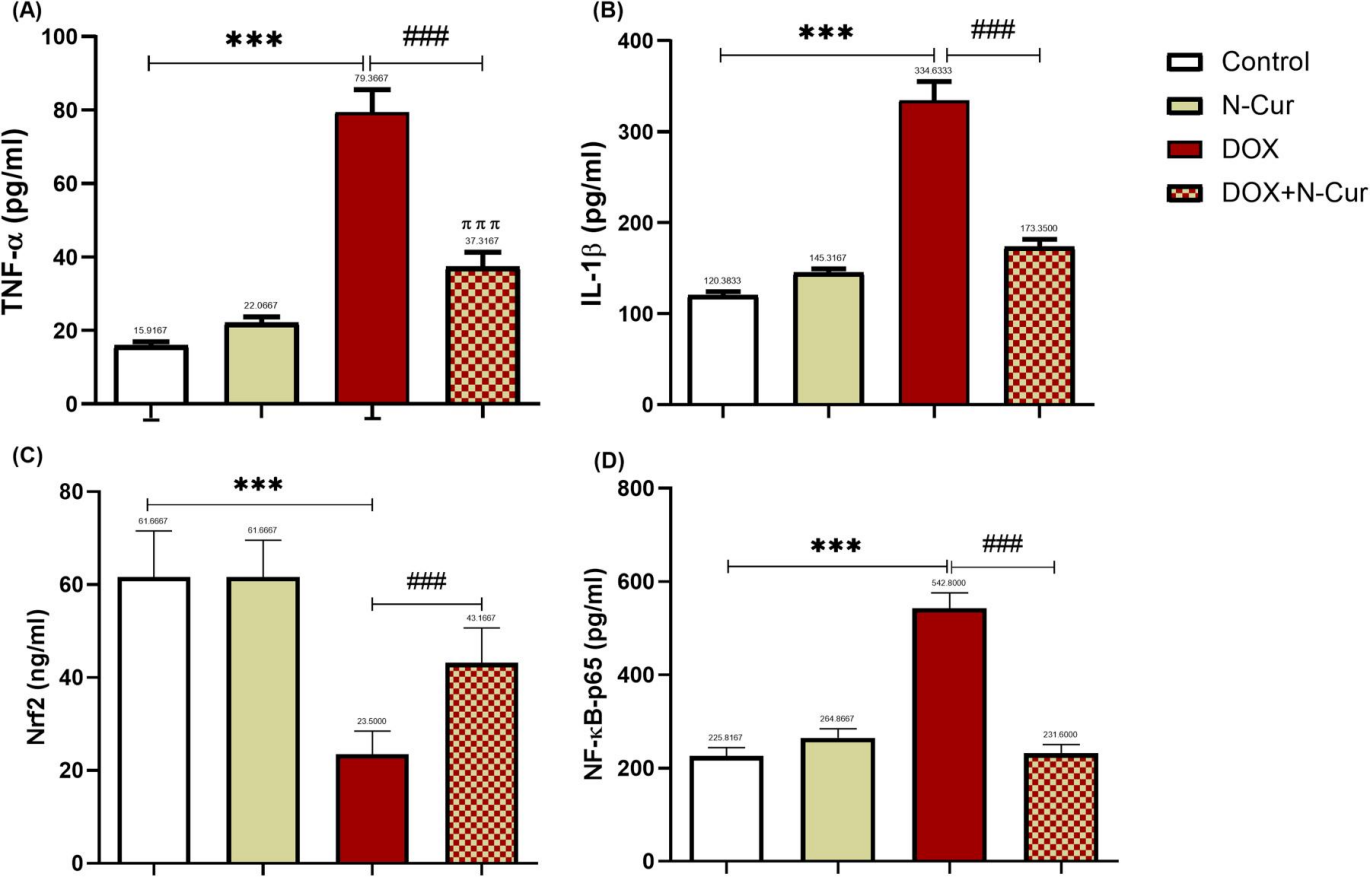
N‐Cur attenuates cardiac levels of TNF‐α (A), IL‐1β (B), Nrf2 (C), and NF‐κB‐p65 (D) in DOX‐ induced cardiac injury. Abbreviations: DOX, doxorubicin; IL‐1β, Interleukin‐1 beta; NF‐κB‐p65, Nuclear factor kappa‐B; N‐Cur, nanocurcumin; TNF‐α, Tumor necrosis factor‐alpha. Data are means ± SE, (*n* = 6). ***p ≤ 0.001 compare to control group, and ^###^ *p* ≤ 0.001 compare to DOX group.

### 3.4 N‐Cur Modulates Wnt/β‐Catenin/GSK‐3 Expression in DOX‐Treated Hearts

Immunohistochemical analysis demonstrated that DOX‐administration significantly disrupted key survival signaling pathways within the heart, an effect that was markedly attenuated by N‐Cur supplementation. In control and N‐Cur‐treated rats, β‐catenin expression was strongly positive in numerous cardiac muscle fibers however, DOX administration severely suppressed this survival signaling, resulting in weak immune positivity. Conversely, co‐administration of DOX and N‐Cur successfully restored β‐catenin levels, as evidenced by a marked increase of the immunoreactivity (Figure 4I). Conversely, the expression of GSK‐3β, a downstream regulator, followed an opposite pattern: control and N‐Cur‐only groups exhibited virtually no immune reaction, whereas DOX administration induced robust GSK‐3β positivity of numerous myocardial cells. Notably, the concurrent administration of N‐Cur with DOX effectively countered this pathological activation, leading to a marked depletion of GSK‐3β immunostaining (Figure 4II).

**Figure 4:**
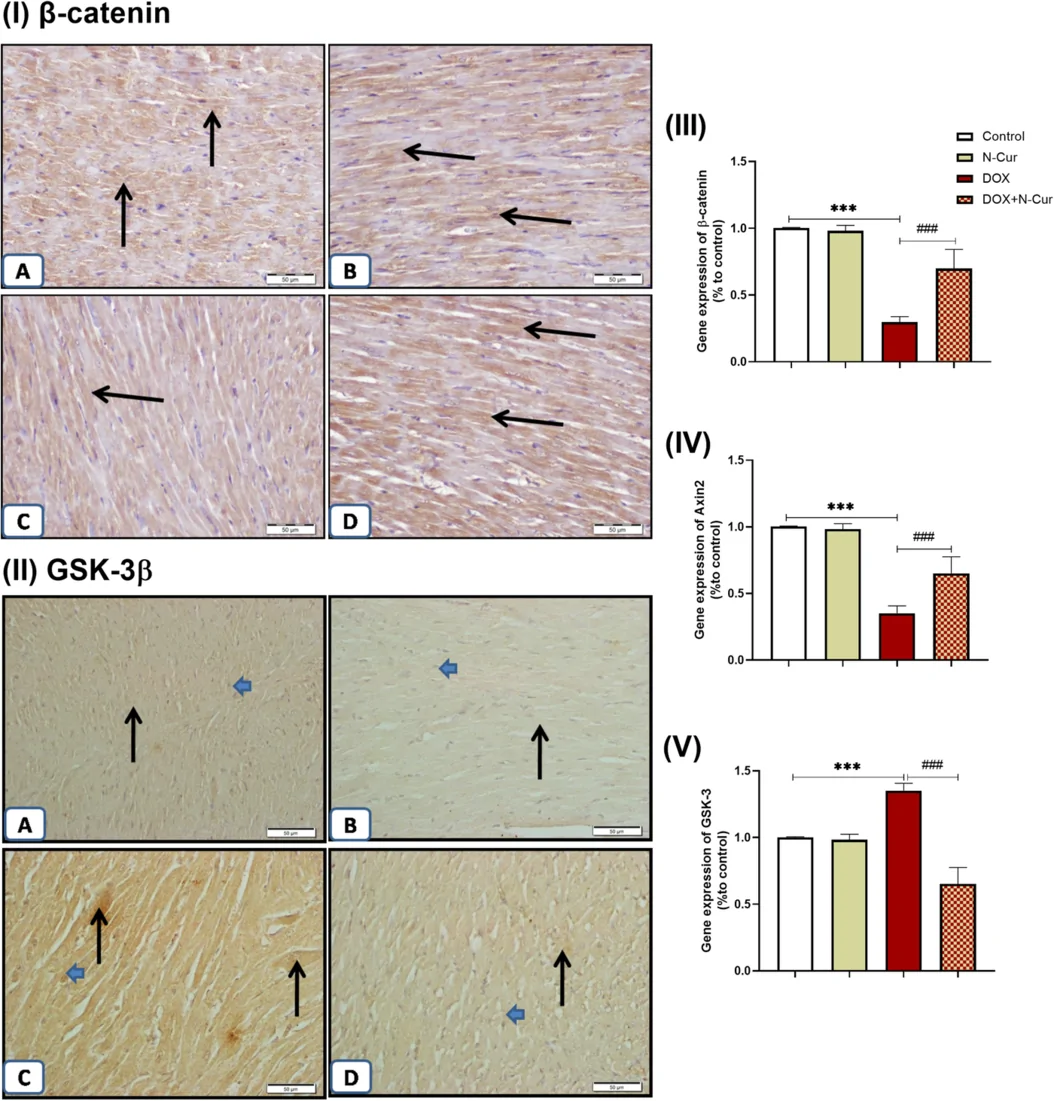
(I) Immuno‐histochemical and RT‐PCR analysis of **β‐catenin** in the heart of rats. Scale bare 50 µm. (A and B) are sections from normal control and rat received N‐Cur, respectively, showing positive immune reaction of cytoplasm of many cardiac cell fibers (arrows). (C) section from rat received DOX, showing few myocardial muscle fibers that weakly stained with β‐catenin (arrow). (D) heart section from rat administered DOX and N‐Cur showing marked increase of immunostained myocardial cell (arrows). (II) Immuno‐histochemical analysis of **GSK‐3β** Rats heart. Scale bare 50 µm. (A and B) are heart sections from normal control and rat received N‐Curcumin, respectively, showing almost absence of immune reaction of both the cytoplasm of cardiac fibers (arrows) and their nuclei (arrow heads). (C) section from rat received DOX, showing many myocardial cells cytoplasm strongly immuno‐stained (arrows) and also their nuclear are positively stained (arrow head. (D) heart section from rat administered DOX and N‐Cur showing marked decrease of immune reaction of both myocardium cytoplasm (arrows) and nuclei (arrow head). RT‐PCR was used to assess gene expression of β‐catenin (III), Axin2 (IV), and GSK‐3 (V), in DOX‐induced cardiotoxicity in rats with and without N‐Cur treatment. Data are means ± SE, (*n* = 6). ***p ≤ 0.001 compare to control group, and ^###^ *p* ≤ 0.001 compare to DOX group.

Consistent with the immunohistochemical protein profiles, RT‐PCR analysis revealed that DOX administration significantly altered the transcriptional activity of key Wnt/β‐catenin signaling components, whereas N‐Cur co‐treatment effectively prevented these molecular disruptions. Specifically, Wnt/β‐catenin (CTNNB1) mRNA paralleled its protein expression, exhibiting high basal gene expression in control and N‐Cur‐only hearts, a marked downregulation following DOX exposure, and a significant transcriptional restoration upon N‐Cur co‐administration (Figure 4III). Conversely, GSK‐3β mRNA exhibited low baseline levels in control and N‐Cur groups, experienced a sharp transcriptional upregulation in response to DOX, and was significantly repressed by concurrent N‐Cur treatment (Figure 4V). Furthermore, mRNA expression of Axin2—a direct downstream target and negative feedback regulator of Wnt/β‐catenin signaling—was robust in control and N‐Cur hearts but profoundly suppressed by DOX, confirming the loss of β‐catenin transcriptional activity; notably, N‐Cur co‐treatment preserved Axin2 transcript levels near physiological baseline (Figure 4IV).

## 4 Discussion

Dox‐induced cardiotoxicity continues to present a significant challenge in clinical oncology, largely attributable to its multifaceted molecular pathology [21]. Accumulating evidence indicates that excessive oxidative stress [22], intense mitochondrial impairment [23], and abnormal cell‑survival signaling networks collectively contribute to the progression of DOX‑induced cardiotoxicity [2]. The Wnt/β‑catenin–GSK‑3β signaling pathway has increasingly been recognized as an essential determinant of cardiomyocyte outcome in DOX‐ induced cardiotoxicity [5]. The large amount of ROS produced during DOX treatment results in mitochondrial dysfunction and activates GSK‐3β, which in turn leads to the phosphorylation and degradation of β‐catenin [9]. As a result, when canonical Wnt/β‐catenin signaling is suppressed, the transcription of genes that are involved in the survival, proliferation and tissue repair of cardiomyocytes is reduced, thus increasing apoptosis and causing myocardial injury [10, 24, 25]. Furthermore, oxidative stress can increase the expression of Wnt antagonists, such as DKK1, and promote activation of non‐canonical Wnt signaling, thereby worsening cardiac damage [3, 10]. On the other hand, restoring redox homeostasis helps maintain β‐catenin signaling, highlighting how closely oxidative stress and Wnt signaling are linked in regulating cardiac cell survival [26, 27]. The present study highlights that disruption of this axis in DOX‐induced cardiac injury and provides a mechanistic basis for the cardioprotective potential of N‐Cur.

Several previous studies indicate that DOX suppresses Wnt/β‐catenin signaling, as reflected by reduced β‐catenin stability and transcriptional activity [10, 11, 28]. This suppression is partly mediated through upregulation of Wnt antagonists such as DKK1 and enhanced activity of GSK‐3β, which promotes β‐catenin phosphorylation and proteasomal degradation [5]. As β‐catenin regulates genes involved in cell survival, anti‐apoptotic signaling, and mitochondrial integrity, its suppression demolishes key cytoprotective signals, thereby promoting cardiomyocyte apoptosis and progressive functional impairment [29, 30]. In this context, N‐Cur represents a promising multi‑target therapeutic agent. While curcumin is limited by poor bioavailability, nanoformulation significantly improves its pharmacokinetic activity, enabling more effective intracellular uptake and extending its duration of action [31, 32]. Our study results indicate that N‐Cur treatment restored Wnt/β‐catenin signaling, which has been shown to attenuate DOX‐induced cardiac injury.

GSK‐3β plays an essential role in this regulatory pathway. Beyond its canonical role in β‐catenin degradation, GSK‐3β integrates signals from oxidative stress, inflammatory mediators, and mitochondrial pathways. Our study results reveal that DOX induces GSK‐3β activation, consistent with a previous study by Liao et al., who found that GSK‐3β stimulation exacerbates mitochondrial permeability transition and facilitates apoptosis [33]. Notably, GSK‑3β also interacts with additional cardioprotective signaling pathways, mainly the PI3K/Akt pathway [34, 35], suggesting that its disturbance compromises several cardioprotective mechanisms.

According to our study results, N‐Cur has been shown to inhibit GSK‐3β activity, thereby preventing β‐catenin degradation, promoting its cytoplasmic accumulation, and restoring Wnt/β‐catenin signaling in DOX‐exposed cardiomyocytes, findings that agree with Sarawi et al. regarding neurotoxicity [36] and GSK‐3β inhibition in cardiac hypertrophy, as shown by Nehra et al. [37]. Oxidative stress is a deep‐rooted suppressor of Wnt signaling, and ROS generation during DOX treatment further destabilizes β‐catenin and activates GSK‐3β, thereby suppressing Wnt signaling. In addition, DOX‐induced oxidative stress alters the Akt/GSK‐3β pathway, promoting apoptosis and mitochondrial permeability transition [38]. It is worth noting that β‐catenin immunoreactivity was primarily observed in the cytoplasm of cardiomyocytes, with no detectable nuclear staining in any experimental group. Although nuclear accumulation of β‐catenin is considered a characteristic feature of canonical WNT pathway activation, its translocation is highly dynamic and often transient, making it difficult to detect by conventional immunohistochemistry at a single time point [39]. Moreover, in mature cardiac tissue, the majority of cellular β‐catenin is strongly sequestered at the intercalated disks and at the cell‐cell junctions through structural interactions with N‐cadherin [40]. Furthermore, under pathological conditions such as myocardial ischemia or cellular stress, the canonical transcriptional programs can be maintained through crosstalk with auxiliary survival pathways, such as the PI3K/Akt axis, thereby altering the kinetic clearance and structural masking of nuclear components [41]. Thus, the increased β‐catenin expression seen after N‐Cur treatment, together with the corresponding decrease in GSK‐3β expression, indicates that the canonical Wnt/β‐catenin signaling axis has been activated. Nevertheless, these results are confirmed by the assessment of the downstream target gene Axin2, using Rt‐PCR.

Our study shows that N‐Cur exerted indirect regulatory effects on the Wnt/GSK‐3β axis through its antioxidant and anti‐inflammatory properties. There is a reciprocal crosstalk between Nrf2 and NF‑κB that constitutes a fundamental regulatory axis in the molecular pathology of DOX‑induced cardiotoxicity [42]. Myocardial cells encompass Nrf2 [43]. Nrf2 inhibits NF‑κB activation by lowering intracellular ROS levels and limiting oxidative stress–driven inflammatory signaling [44]. In contrast, sustained NF‑κB activation suppresses Nrf2‑dependent transcription, diminishing the cell's antioxidant capacity. Consequently, during DOX exposure, this regulatory balance shifts toward NF‑κB‑mediated inflammation and away from Nrf2‑driven protection [42, 44, 45].

In our study, N‐Cur activated endogenous antioxidant defenses, particularly the Nrf2 axis, and effectively reduced MDA levels while restoring GSH and SOD activities. These results collectively diminish the cellular oxidative stress burden, thereby facilitating reactivation of Wnt signaling. These results are consistent with previous experiments on FFA, which induces cardiac injury and cytopathy [37, 38, 39, 46]. In parallel, N‐Cur suppressed NF‑κB–mediated inflammatory cascades and reduced TNF‐α and IL‐1β levels. These activities come in accordance with Ma et al., and Huang et al., who stated that suppression of NF‑κB and the use of N‐Cur mitigates the inhibitory cross‑talk that otherwise destabilizes β‑catenin activity, collectively supporting the restoration of Wnt/β‑catenin pathway integrity under oxidative and inflammatory stress [40, 41]. Collectively, our findings revealed that N‐Cur mitigates DOX‑induced cardiotoxicity by concurrently modulating oxidative stress and inflammatory signaling. Consequently, this coordinated modulation of Wnt/GSK‐3β indicates that N‐Cur does not exert its effects through a single molecular target but instead reinstates a broader, interconnected signaling network that is fundamental for fighting Dox‐induced cardiotoxicity. In addition, Wnt/GSK‐3β modulation involves maintaining mitochondrial integrity. In our study, inhibiting GSK‐3β with N‐Cur stabilizes mitochondrial membranes, attenuates mitochondrial ROS production, and enhances ATP production. These effects are consistent with the neuroprotection properties of curcumin in previous experimental models [42, 43] which are enhanced in its nanoformulated form.

Cardiac troponin I (cTnI) is widely recognized as a highly sensitive and specific biomarker of myocardial injury [44]. In the present study, DOX‑treated rats exhibited marked elevations in cTnI and CK‑MB, accompanied by histopathological evidence of substantial cardiomyocyte damage, including extensive myocardial degeneration and prominent inflammatory cell infiltration. These results are consistent with the previous study of Bertinchant etal on DOX‐induced cardiomyopathy [44] and Hanifah etal, respectively [45]. In contrast, N‐Cur administration significantly ameliorated these structural alterations and restored cardiac enzyme levels toward normal values, in agreement with previous investigations of Shati et al demonstrating the cardioprotective actions of curcumin against DOX‑induced cardiac toxicity [46]. Extrapolating from these findings, using N‐Cur in our experiment is likely to exert a more pronounced effect.

In conclusion, the Wnt/β‐catenin–GSK‐3β axis plays a central role in DOX‐induced cardiotoxicity, contributing to oxidative stress, inflammation, and mitochondrial dysfunction pathways. N‐Cur can restore homeostasis of this axis and alleviate cardiac injury.

## 5 Limitations

There are certain restrictions on our research. Since we only employed one acute dosage of DOX, cumulative clinical regimens are not recapitulated. Furthermore, since only one N‐Cur dose was used, the dose‐response investigation was not carried out. To translate these findings into therapeutically useful cardioprotective treatments, future research will include β‐catenin nuclear translocation, TCF/LEF transcriptional activity, selective Wnt/β‐catenin agonists or antagonists, GSK‐3β inhibitors, and gene silencing or overexpression techniques. To further clarify its therapeutic efficacy, chronic DOX exposure will be assessed, functional cardiac evaluation (e.g., echocardiography) will be performed, and larger cohorts and multiple dosing regimens will be evaluated.

## Author Contributions

**Noha A. Alshuwayer:** resources, funding acquisition, writing – original draft. **Qamraa H. Alqahtani:** writing – original draft, methodology. **Marwa H. Hussein:** writing – original draft, writing – review and editing, resources. **Raeesa Mohammed:** methodology, resources, funding acquisition, writing – original draft. **Abdulaziz Siyal:** methodology. **Iman H. Hasan:** conceptualization, writing – original draft, writing – review and editing, methodology, software, formal analysis, project administration, supervision.

## Conflicts of Interest

The authors declare no conflicts of interest.

## Institutional Review Board Statement

The experiment was carried out in accordance with the guidelines outlined in the National Institutes of Health (NIH) publication No. 85‐23, revised in 2011. Additionally, it received approval from the research ethics committee at King Saud University (ethical reference number KSU‐SE‐24‐68).

## Data Availability

Data generated during this study are available upon request from the corresponding author.
